# The Platelet-Derived Growth Factor Pathway in Pulmonary Arterial Hypertension: Still an Interesting Target?

**DOI:** 10.3390/life12050658

**Published:** 2022-04-29

**Authors:** Julien Solinc, Jonathan Ribot, Florent Soubrier, Catherine Pavoine, France Dierick, Sophie Nadaud

**Affiliations:** 1 INSERM, Institute of Cardiometabolism and Nutrition (ICAN), Sorbonne Université, UMR_S1166, F-75013 Paris, France; julien.solinc@gmail.com (J.S.); jonathan.ribot@inserm.fr (J.R.); florent.soubrier@sorbonne-universite.fr (F.S.); catherine.pavoine@inserm.fr (C.P.); 2 Lady Davis Institute for Medical Research, McGill University, Montreal, QC H3T 1E2, Canada; france.dierick@mail.mcgill.ca

**Keywords:** PDGF, PDGFR, smooth muscle cells, fibroblast, pulmonary arterial hypertension, vascular remodeling, Imatinib

## Abstract

The lack of curative options for pulmonary arterial hypertension drives important research to understand the mechanisms underlying this devastating disease. Among the main identified pathways, the platelet-derived growth factor (PDGF) pathway was established to control vascular remodeling and anti-PDGF receptor (PDGFR) drugs were shown to reverse the disease in experimental models. Four different isoforms of PDGF are produced by various cell types in the lung. PDGFs control vascular cells migration, proliferation and survival through binding to their receptors PDGFRα and β. They elicit multiple intracellular signaling pathways which have been particularly studied in pulmonary smooth muscle cells. Activation of the PDGF pathway has been demonstrated both in patients and in pulmonary hypertension (PH) experimental models. Tyrosine kinase inhibitors (TKI) are numerous but without real specificity and Imatinib, one of the most specific, resulted in beneficial effects. However, adverse events and treatment discontinuation discouraged to pursue this therapy. Novel therapeutic strategies are currently under experimental evaluation. For TKI, they include intratracheal drug administration, low dosage or nanoparticles delivery. Specific anti-PDGF and anti-PDGFR molecules can also be designed such as new TKI, soluble receptors, aptamers or oligonucleotides.

## 1. Introduction

**Figure 1:**
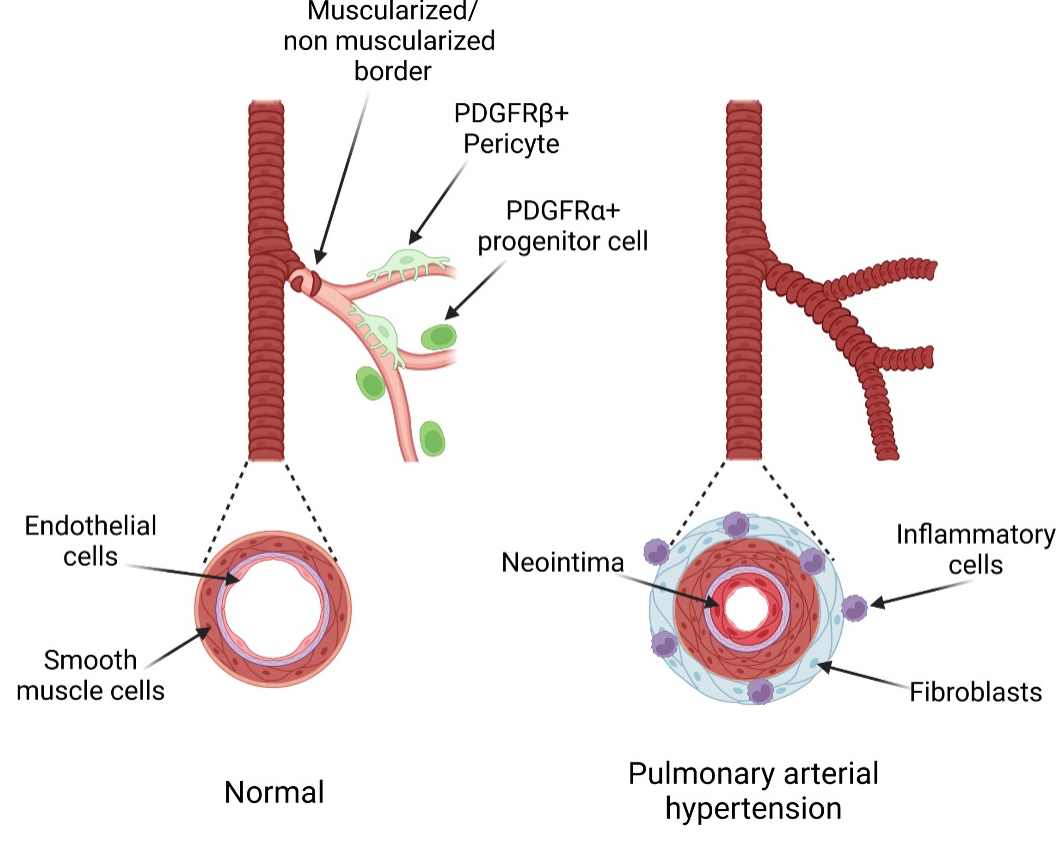
Pulmonary arterial hypertension-associated vascular remodeling. Pulmonary arterioles are mostly non muscularized at basal state. They are in close proximity with PDGFRβ+ pericytes, PDGFRα+ progenitor cells and fibroblasts. In the course of the disease, the small arterioles are covered with new smooth muscle cells formed from smooth muscles cells, pericytes and PDGFRα+ progenitor cells. The media of muscularized arteries thickens and myofibroblasts proliferate and migrate forming a neointima. Fibroblasts and immune cells accumulate in the perivascular zone.

Pulmonary arterial hypertension (PAH) is a devastating disease with no cure yet. It is characterized by vascular remodeling, vessel contraction, endothelial dysfunction, in situ thrombosis, fibrosis and inflammation. During the course of the disease, new vascular smooth muscle cells are produced, resulting in neomuscularization and media thickening of pulmonary arteries (Figure 1). Myofibroblasts accumulate forming a neointima which progressively occludes pulmonary arterioles. Complex angioproliferative occlusive regions called plexiform lesions are also present. Inflammatory cells infiltration can be observed in perivascular and adventitial areas. Increased pulmonary resistance leads to right ventricular failure and ultimately death in the absence of cardiopulmonary transplantation. The current therapeutic options mainly aim at inhibiting vascular contraction with nitric oxide donors, prostacyclin and endothelin receptor inhibitors. Investigations for new pathways involved in PAH development and its associated occlusive vascular remodeling have been ongoing for the past 30 years and led to several important discoveries (e.g., BMPR2, ALK1, KCNK3, TBX4 …) [1,2]. Among these, the role of the platelet-derived growth factor (PDGF) pathway was demonstrated both in experimental models and in patients [3,4]. The disappointing results of the clinical trials with the PDGF receptor (PDGFR) inhibitor Imatinib, showing reduced pulmonary vascular resistance but association with serious adverse events [5], slowed down the interest in this pathway. However, extensive data show the important impact of this pathway on pulmonary vascular remodeling. In addition, a recent study highlighted PDGF-D as a new candidate risk gene for adult-onset idiopathic PAH [6]. The development of novel therapeutic approaches or new drugs, allowing to specifically reach the lung or to inhibit particular members of the PDGF family, may in the future allow the detrimental effects of the anti-PDGFR therapy to be reduced while improving the pulmonary vascular structure and function of patients. In this review, we will present the PDGF/PDGFR pathway and the data demonstrating its major role in controlling pulmonary vascular remodeling and pulmonary hypertension development. We will discuss the future therapeutic options targeting this pathway to improve the PAH patients’ condition.

## 2. PDGF Ligands and Their Receptors PDGFR

**Figure 2:**
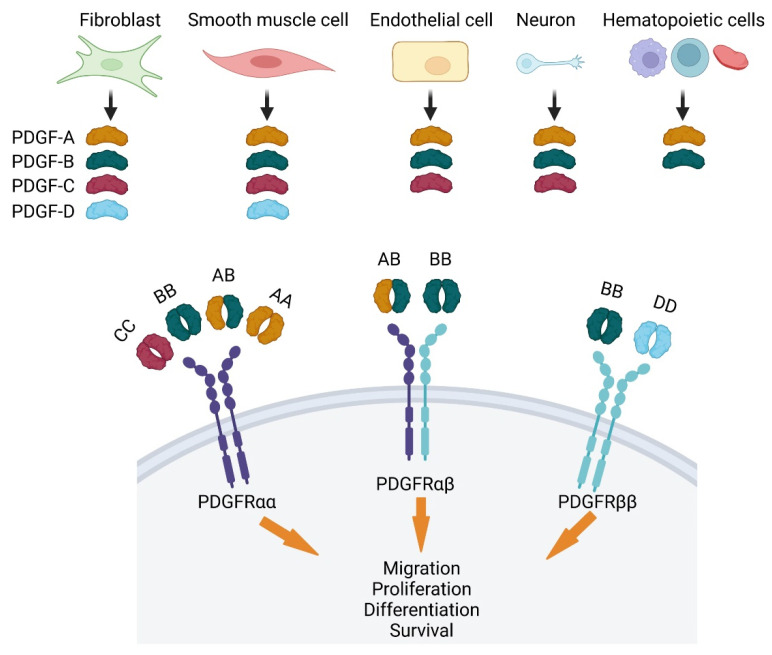
PDGF production and PDGF receptors specificity in and by pulmonary cells. PDGF are produced as four isoforms (A, to D) which are present as 4 homodimers and 1 heterodimer (PDGF-AB). Fibroblasts express the 4 isoforms of PDGF, endothelial cells and neurons express PDGF-A to C and platelet, macrophages and lymphocytes express PDGF-A and B. The PDGF receptor type α (PDGFRα) binds PDGF-A, -B and -C when PDGFRβ binds PDGF-B and -D. Both receptors dimerize upon PDGF binding leading to homo or heterodimers with different binding specificities. PDGF signaling involves multiple pathways and lead to cell proliferation, migration, differentiation and survival.

Platelet-derived growth factor (PDGF) was discovered in 1970 as a platelet-dependent serum factor that was known to be released upon degranulation, stimulating the proliferation of fibroblasts, arterial smooth muscle cells and glial cell [7]. In the following years, PDGFs were better characterized, and they are now known as a family of four cystine-knot-type growth factors (PDGF-A, -B, -C and -D) that reside on chromosomes 7, 22, 4 and 11 in humans, and chromosomes 5, 15, 3 and 9 in mice. The four PDGFs form homodimers PDGF-AA, BB, CC, DD and one heterodimer, PDGF-AB (Figure 2). They control the growth of connective tissue cells such as fibroblasts and smooth muscle cells [8]. By acting on mesenchymal cells, PDGFs regulate embryonic development in particular the formation of vessels and organs [8]. There are two types of receptors for PDGFs, PDGFRα and PDGFRβ, which belong to the class III receptor tyrosine kinases. They encode a transmembrane protein with an extracellular ligand binding domain and an intracellular tyrosine kinase domain. Each of the two PDGF molecules within a PDGF dimer binds one molecule of PDGFR. Hence, ligand binding induces dimerization of PDGFRs, which are monomeric prior to PDGF exposure. PDGFRα signaling controls gastrulation and the development of several organs such as lung, intestine, skin, testis, kidney, bones, and neuroprotective tissues. PDGFRβ signaling is an essential regulator of early hematopoiesis and blood vessel formation [9]. Interaction between PDGFs and their receptors plays a major role during development and their expression is finely tuned in adulthood. PDGF-A and PDGF-B are 60% homologous in their amino acid sequence [7]. They are activated by proteolytic cleavage in the cell before secretion. The main enzyme responsible for activating PDGF-A is a furin convertase, whereas it remains unknown for PDGF-B [10]. Conversely, PDGF-C and PDGF-D are activated by extracellular proteolytic cleavage [11]. PDGF-C can be activated by plasmin, tissue plasminogen activator and urokinase plasminogen activator [12], whereas PDGF-D can only be activated by plasmin or the urokinase plasminogen activator [13,14].

The PDGF receptors are composed of an extracellular ligand recognition domain, a single transmembrane helix to transduce the signal and a tyrosine kinase effector domain that responds to the extracellular activation and undergoes phosphorylation to induce downstream signaling events. PDGF binding activates the receptor kinase activity. Tyrosine phosphorylation of the receptor itself and of other substrates triggers intracellular signaling cascades that are essential to evoke cellular responses such as migration and proliferation [15]. PDGFRα and PDGFRβ contain 10 and 11 known tyrosine residues, respectively, which can dock signaling molecules upon autophosphorylation. Several families of Src homology region 2 (SH2)-domain containing molecules (adaptors or enzymes) have been shown to bind different phosphorylated residues in those receptors [16]. The main PDGF-activated pathways are (1) PI3K/AKT/mTOR which controls cellular survival, growth, proliferation, and metabolic activity, (2) ras/MAPK involved in cell survival, proliferation, differentiation and migration regulation, and (3) PLCγ/PKC which regulates intracellular calcium mobilization, proliferation and migration [8,10,12,17]. Those receptors can also stimulate Jun-Activated Kinase (JAK) and then STAT proteins (Signal Transducers and Activators of Transcription) which act as transcription factors upon translocation into the nucleus [18]. 

Ligand mediated dimerization and activation of the PDGFR kinase also hastens internalization mostly through a clathrin-mediated endocytosis followed by degradation leading to signal attenuation [19]. Hence, the PDGFRα and PDGFRβ half-lives are reduced from 2 h in resting cells to 5 and 30 min following PDGF binding [20]. The two forms of PDGFR exhibit binding specificities toward the PDGF isoforms (Figure 2). PDGFRα binds the PDGF-A, -B and -C chains with high affinities, and PDGFRβ binds the PDGF-B and PDGF-D chains with high affinities as well. Consequently, PDGFRα is activated by PDGF-AA, PDGF-BB, and PDGF-CC homodimers and PDGF-AB heterodimer. PDGFRβ can only be activated by PDGF-BB and PDGF-DD [8,10]. The PDGFRαβ heterodimerization can be induced by the PDGF-BB homodimer or by the PDGF-AB heterodimer. PDGF-independent modes of receptor activation can also occur both in pathological and physiological settings. The best-studied form of PDGF-independent activation of PDGFRs is the indirect mode, which is driven by non-PDGFs growth factors outside of the PDGF family. Binding of these growth factors to their own receptors leads to NADPH oxidase-driven reactive oxygen species generation which stimulate Src family kinases (SFKs) to phosphorylate monomeric PDGFRα. This mechanism provides an explanation for how cancer cells survive and proceed to drive pathogenesis in the absence of pro-survival PDGF related factors [21]. Prolonged activation of PDGFRα can be observed and is supported by two mechanisms. First, indirect activation involves monomeric PDGFRαs forms which do not trigger self-destruction of the receptor as for activated dimeric PDGFRα forms. This can involve the binding of VEGF (vascular endothelial growth factor) which competes with PDGF. Second, indirect activation of PDGFRα engages a feed-forward loop that perpetuates activation of monomeric PDGFRα [22].

## 3. PDGF Effects on Pulmonary Cells

### 3.1. PDGF and Pulmonary Endothelial Cells 

**Figure 3:**
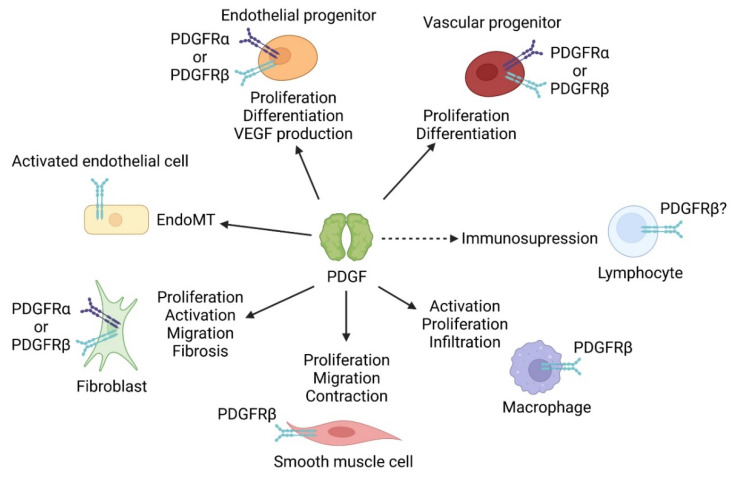
PDGF regulation of pulmonary cells function. Most cells involved in PAH development are regulated by PDGF. Several cell types express either PDGFRα or PDGFRβ according to different studies. SMC and macrophages only express PDGFRβ. PDGFRβ+ as well as PDGFRα+ progenitor cells are regulated by PDGF during PH development in experimental models. PDGF induces proliferation and differentiation of vascular PDGFRβ+ progenitor cells whereas it only induces proliferation of perivascular PDGFRα+ progenitor cells. Fibroblasts expressing PDGFRα or PDGFRβ are activated by PDGF leading to proliferation, migration, and fibrotic activity. Activation of PDGFRβ leads to SMC proliferation and migration but also to their contraction. Expression of PDGFR by lymphocyte and dendritic cells has not been demonstrated; yet their activity was shown to be regulated by PDGF-BB. Endothelial-to-mesenchymal (EndoMT) transition may also be induced by PDGF.

PDGF receptors are not present on mature endothelial cells but could be expressed by haemangioprecursor cells expressing PDGFRβ, and endothelial progenitor cells. PDGF ligands stimulate endothelial progenitor cells proliferation and differentiation into endothelial cells [23] and their production of VEGF through KLF4 (Kruppel-like factor 4) activation [24]. Hence, both receptors are necessary for correct vascular development during embryogenesis and genetic deletions of PDGFR and PDGFs result in vascular defects. Thus, the PDGF pathway is a major regulator of vessels formation and maintenance in the course of vasculogenesis, de novo vascular network formation in the embryo, and during angiogenesis, formation of new capillaries from pre-existing vessels. During the sprouting process, the new vessels are stabilized through the recruitment of pericytes or smooth muscle cell (SMC). Endothelial-derived PDGF signaling stimulates mural cells recruitment, migration and vessel coverage and stabilization [25,26,27]. Endothelial-specific PDGF-BB knock-out results in a similar phenotype as PDGFRβ KO, indicating that paracrine signaling between the endothelium and pericytes is required in the process of pericyte recruitment [27]. Indeed, PDGF-BB stabilizes pericytes interaction with endothelial cells and reduces endothelial proliferation and aberrant angiogenesis by regulating pericyte-endothelial crosstalk in newly formed vessels [28]. An endothelial-to-mesenchymal (EndoMT) transition process has been demonstrated during pulmonary artery remodeling in PAH. Some studies suggest that endothelial cells activation by PDGF-A and PDGF-B could participate in this transition [29]. One could speculate that a prior activation of endothelial cells leading to PDGFR expression may be necessary (Figure 3).

### 3.2. PDGF and Pulmonary Arterial Smooth Muscle Cells

**Figure 4:**
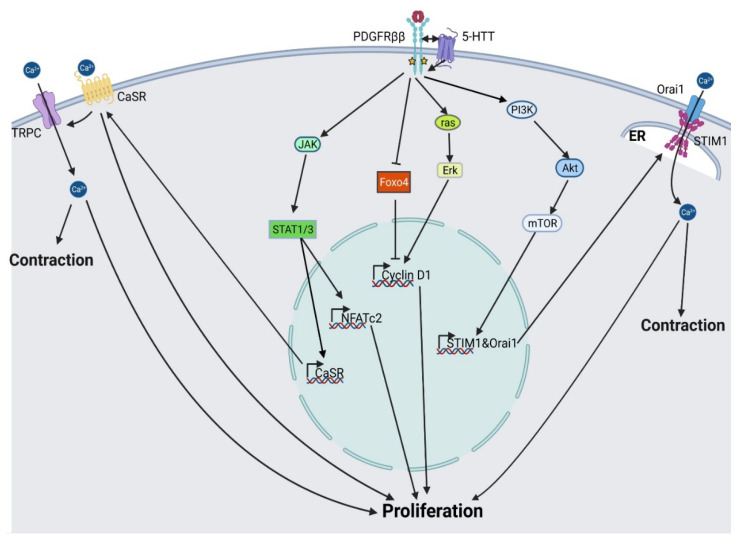
The PDGFRβ pathway in pulmonary smooth muscle cells. Binding of PDGF to PDGFRβ and its subsequent autophosphorylation have been shown to trigger multiple signals in pulmonary smooth muscle cells. It activates the ras/Erk pathway promoting Cyclin D1 transcription, a major regulator of the cell cycle. The Akt/mTOR pathway was also demonstrated to induce STIM1 and Orai1 expression. The complex Orai1/STIM1 is responsible for store-operated calcium entry into PASMC leading to contraction and proliferation. The PDGF pathway stimulates JAK and the STAT transcription factors leading to enhanced expression of NFATc2 and CaSR which are both pro-proliferative. CaSR also activates TRPC channels calcium entry which provokes contraction and proliferation. PDGF stimulation increases Foxo4 ubiquitination and degradation. Foxo4 is a transcription factor that downregulates Cyclin D1 expression, a process alleviated upon PDGF activation. PDGFRβ activity is enhanced by direct interaction with the serotonin transporter 5-HTT which is increased during PAH. ER, endoplasmic reticulum; STIM1, stromal interaction molecule 1; ORAI1, ORAI Calcium Release-Activated Calcium Modulator 1; CaSR, extracellular calcium sensing receptor; TRPC, Transient Receptor Potential Cation Channel; 5-HTR, Serotonin receptor; JAK, Janus kinase; STAT, signal transducer and activator of transcription proteins; NFATc2, Nuclear factor of activated T-cells, cytoplasmic 2; ras, rat sarcoma virus; Erk, Extracellular signal-regulated kinase; Pi3k, Phosphoinositide 3-kinase; Akt, Protein kinase B; mTOR, Mechanistic Target Of Rapamycin Kinase; Foxo4, Forkhead box protein O4.

Smooth muscle cells are key players in pulmonary hypertension-associated vascular remodeling [30]. In this pathological setting, SMCs switch from their physiological contractile phenotype to a pathophysiological proliferative and synthetic phenotype and migrate into the intima. Differentiated SMCs mostly express PDGFRβ, rarely PDGFRα [31], and PDGF-BB released from aggregating platelets and endothelial cells at sites of vascular injury is a major inducer of their proliferation and migration [8] (Figure 3). PDGFRβ signals mainly through the MAPK (Mitogen-Activated Protein Kinase) and the Akt pathways which tightly control cell survival, growth, proliferation and metabolic activity [10] (Figure 4). PDGFRβ activation of Akt and P38 is increased by its interaction with the serotonin receptor and this association is stimulated upon PDGF binding [32]. PDGFRβ also activates the JAK and STAT1/3 pathway leading to increased expression of NFATc2 and CaSR (extracellular calcium sensing receptor) and subsequent SMC proliferation. Both factors are found increased in PASMC from iPAH patients [33,34,35]. PDGFRβ stimulation leads to FOXO4 degradation which together with Erk activation induce Cyclin D1 expression and cell proliferation [36].

PDGF not only affects PASMC proliferation, but it also regulates PASMC contractility in pulmonary hypertension. PDGFRβ activation by PDGF-BB was demonstrated to provoke PASMC contraction of pulmonary arteries [37]. This effect was mediated by the generation of prostaglandins, the increase in calcium and cAMP, the activation of MAPK or PI3K/AKT/mTOR signaling and actin remodeling [37]. In particular, PDGFRβ-induced activation of the PI3K/AKT/mTOR pathway enhances expression of STIM1 (Stromal Interaction Molecule 1) and Orai1 (ORAI Calcium Release-Activated Calcium Modulator 1), two partners which constitute the SOCE (store-operated calcium entry) [38]. In addition, the increased expression of CaSR can stimulate TRPC (transient receptor potential conical) calcium channels and calcium entry [39]. Both STIM1/Orai1 and TRPC regulations lead to increased cytosolic calcium concentration promoting PASMC contraction and proliferation.

### 3.3. PDGF and Pulmonary Fibroblasts

Fibroblasts are defined as resident mesenchymal cells which maintain tissue integrity [40]. They produce extracellular matrix, signaling molecules and are able to transiently adopt a contractile phenotype characterized by α-SMA (α-Smooth Muscle Actin) expression. Fibroblasts may be responsible for neointimal myofibroblasts production and for fibrosis, both processes being regulated by PDGF (Figure 3).

All PDGF isoforms have been implicated in fibrosis development in different organs (kidney, lung) and PDGFR inhibition, using Imatinib, reduces bleomycin-induced fibrosis [41]. Indeed, PDGF is one of the factors involved in fibroblast transition from quiescence to myofibroblasts following tissue injury to facilitate tissue repair. PDGFRα expression is induced during the fibrotic process leading to proliferation of PDGFRα+ fibroblasts [42] which contribute to pathological myofibroblasts formation during bleomycin-induced pulmonary fibrosis [43,44]. Fibroblasts stimulation by PDGF-AA induces P38 phosphorylation and subsequent activation of the mesenchymal differentiation regulator SRF (serum responsive factor) leading to α-SMA expression (activated fibroblast) [45]. This was confirmed as constitutive activation of PDGFRα ultimately led to multiple organs fibrosis including lung [46]. However, we observed in this model that cell proliferation takes place long prior to collagen deposition [31], suggesting that other signals may be responsible for fibroblasts activation and collagen production. Interestingly, intratracheal injection of PDGF-BB for 3 days induced peribronchial and perivascular spindle cell proliferation accompanied by collagen deposition [47]. Since PDGF-BB activates both receptors, this result suggests that PDGFRα activation could be responsible for fibroblasts accumulation when PDGFRβ activation could drive them to produce more collagen. Accordingly, transgenic mice with a lung-specific PDGF-C overexpression (targeting PDGFRα) develop massive mesenchymal cell hyperplasia and die from respiratory insufficiency immediately after birth [48]. However, Green et al separated lung PDGFRα+ cells into myofibroblasts (CD29+) and matrix fibroblasts (CD34+). Matrix fibroblasts differentiation was dependent on PDGFRα signaling while myofibroblast differentiation was not [49]. In line, PDGFRβ+ mesenchymal cells were recently demonstrated to be a major source of myofibroblasts during bleomycin-induced lung injury [50]. Thus, fibroblast regulation by PDGF is complex and specific for different subpopulations. 

### 3.4. PDGF and Pulmonary Vascular Smooth Muscle Progenitor Cells

Adult pulmonary vascular progenitor cells have been characterized by several teams [51]. Pericytes and primed smooth muscle cells express PDGFRβ [52] whereas adventitial and perivascular progenitor cells express PDGFRα [31,53] (Figure 1 and Figure 3). Pericytes are extensively studied in numerous organs for their response to PDGF in normal and pathological settings. Pericytes play a pivotal role in angiogenesis and contribute to vessel formation, remodeling and stabilization. Among these, PDGF-B/PDGFRβ signaling pathway is important because of its involvement in pericyte proliferation, survival, and attachment [54]. As stated before, endothelial PDGF-BB production is a major inducer of pericytes recruitment and is necessary for normal angiogenesis [25,26,27,28]. Studies in mice showed that PDGF-BB or of PDGFRβ loss led to a severe deficiency in pericyte recruitment causing microvascular damages with endothelial hyperplasia, aberrant vasculature and microaneurysms [55]. Indeed, expression of a mutant PDGF-B, defective for extracellular matrix binding, resulted in disorganized diffuse arteriolar muscularization and in pericytes loss. These mice did not develop PH after exposure to chronic hypoxia despite a marked neomuscularization and the authors suggested that it may be explained by the loose organization of the media [55]. The early expression of PDGFRβ during the specification of pericytes underlines the importance of the PDGF pathway in their formation [56].

Sheikh et al. have identified a specialized population of medial smooth muscle progenitor cells that give rise to arteriolar SMCs in mice under chronic hypoxia [52]. These cells express PDGFRβ and KLF4 together with classic SMC markers and are positioned at the muscular-unmuscular border of each arteriole [52]. During chronic hypoxia, enhanced PDGF-B levels stimulate their clonal expansion and migration to muscularize the downstream arteriole [52]. 

Other resident SMC progenitor cells expressing PDGFRα have been identified in the vicinity of pulmonary vessels [31,53,57]. They express several stem cells markers including PW1, Sca-1, c-kit, CD34 or mesenchymal stem cells markers such as ABCG2. We recently demonstrated that PW1+/PDGFRα+ progenitor cells are actively recruited in human PAH remodeled arteries and in chronic hypoxic mice. Our results establish that PDGFRα activation induces the formation of new PASMCs by enhancing progenitor cells proliferation albeit not their differentiation. The PDGFRα+ cell lineage gives also rise to myofibroblasts during lung fibrosis. The PDGFRα pathway is believed to be responsible for this fibroblastic differentiation [46]. However, our results suggest that PDGFRα stimulation leads to proliferation of progenitor cells and that other signals are necessary to produce myofibroblasts.

This PDGFRα pathway is of major importance during lung development. PDGFRα+ cells contribute to myofibroblasts and lipofibroblasts during lung maturation [58] and this process is dependent on PDGF-AA signaling [59]. It has also been demonstrated that PDGFRα+ mesoderm generates endothelial cells in embryonic stem cells differentiation culture [60]. In this light, it was suggested that PDGFRα+ cells can contribute to form endothelial cells during mouse embryogenesis [61]. However, the role of the PDGF pathway in this process is still unknown. 

### 3.5. PDGF and Inflammatory Cells 

The PDGF pathway not only regulates vascular cells function but can also target inflammatory cells, which are major players in pulmonary hypertension (Figure 3). However, investigations on their regulation by PDGF in the lung are lacking. In addition, most published studies were performed in vitro with cells that may be altered compared with their in vivo counterparts.

Monocytes, Macrophages, dendritic cells, and T cells express PDGFRβ [10]. Monocyte migration was enhanced by PDGF-AB or BB but not by PDGF-AA [62]. In macrophage, PDGFRβ activation induces cholesterol biosynthesis [63]. PDGF-C has also anti-apoptotic, pro-migratory and pro-proliferative effects on macrophages [64,65]. Yet, PDGF-C preferentially binds to PDGFRα whose expression has not been demonstrated in macrophages. 

PDGF-BB may also display immunosuppressive effects. PDGF treatment reduces CD4+ T-cells proliferation either directly [66] or through inducing dendritic cell CLEC2 expression [67]. Moreover, PDGF-BB-treated dendritic cells produce less TNFα and more IL-10.

## 4. Role and Regulation of the PDGF Pathway in PH

PDGF-B expression and production are upregulated in endothelial cells from iPAH patients suggesting a paracrine regulation of PDGFR by PDGF-B [68,69]. In iPAH patients, PDGF-B is also upregulated in macrophages derived from circulating mononuclear cells, which are probably an important source of PDGF in the pathology [70]. Similarly to PAH patients, PDGF-B is upregulated in macrophages of rodents exposed to chronic hypoxia or MCT [70]. Rodents depleted in macrophages by clodronate and exposed to chronic hypoxia develop less severe vascular remodeling and right ventricular systolic pressure whereas specific PDGF-B depletion in macrophages protected against chronic hypoxia-induced remodeling. Hence, macrophages may represent a major source of PDGF-B stimulating PASMC proliferation through PDGFRβ.

PDGF/PDGF receptors expressions are increased in lungs of PAH patients [3,4,71] and their serum PDGF concentrations are significantly higher [71]. Although expression of both receptor isoforms is upregulated in cultured PASMCs from iPAH patients [34,68], we mostly observed PDGFRα in the adventitia of pulmonary arterioles [31] where PDGFRβ levels are also increased [72,73]. In consequence, PDGFRβ signaling in iPAH PASMCs is enhanced and further supported by a longer lasting phosphorylation. The serotonin pathway plays a central role in PAH pathogenesis and increased serotonin transporter expression and activity is associated with enhanced PDGFRβ signaling [33]. The mineralocorticoid regulation of PDGFRβ activity could also be involved in PASMC and fibroblasts activation during PAH as aldosterone can transactivate PDGFR [74] by phosphorylating the receptor [75]. This regulation may participate in the beneficial effect of mineralocorticoid receptor inhibition observed in PH models [76]. PDGFR expression can also be altered in endothelial cells since FGF2, another key signaling pathway activated during PAH, can trigger PDGFRα and β expression at the transcriptional level [77].

The PDGF/PDGFR pathways involvement in PH development has been demonstrated in several studies using different experimental models. Similarly to patients, PDGFRβ expression and activation are increased in rodent exposed to chronic hypoxia or monocrotaline (MCT), two PH models [3]. Expression of all members of the pathway (PDGFRα and β and the 4 PDGF chains) was increased during early chronic hypoxia in mice suggesting an early activation of both receptors in this model [31]. Several studies targeting PDGFR or PDGF confirmed that this pathway is a major regulator of PH development and of pulmonary vascular remodeling. PDGFRs inhibition with Imatinib, which also inhibits c-kit and c-abl, improved MCT-induced PH rat survival. Moreover, PDGFRs inhibition reduced vascular remodeling and right ventricular hypertrophy in both experimental models [3]. In chronic hypoxia, Inhibition of PDGFRβ signaling also prevented PH development in chronic hypoxia mice [78], whereas specific PDGFRα inhibition reduced pulmonary vascular muscularization only at an early stage [31]. In line with these results, expression of a mutated form of PDGF-B defective for extracellular matrix retention prevented PH development after chronic hypoxia and led to a dispersed pulmonary muscularization. Conversely, PDGFRβ constitutive activation was not sufficient to induce PH but this transgenic model was more sensitive to chronic hypoxia and developed stronger pulmonary vessels muscularization [79]. Unlike PDGFRβ, constitutive activation of PDGFRα, as well as PDGF-A administration, induced PH but solely in male mice [31]. Intratracheal administration of PDGF-BB in rats lead to the hyperproliferation of PASMCs and increased lung fibrosis [47]. 

Several experiments showed various effects of PDGFRβ activation on vascular remodeling. Its overactivation showed a direct impact on PASMC proliferation, thus participating in the pulmonary vessels muscularization. However, other indirect effects of PDGFRs could be involved in regulating vascular structure and function. In PAH patients, PDGFRs and CaSR (extracellular calcium receptor) overexpression are associated and lead to an upregulated calcium-dependent signalization, participating in PASMC proliferation. This CaSR increase is inhibited by PDGFRα or PDGFRβ downregulation using specific siRNAs in patients PASMC. In turn, CaSR inhibition or deletion protects against experimental PH development [33,39,80]. PAH patients show elevated levels of serotonin which could participate in increasing PDGF signaling. A direct interaction between the serotonin transporter (5-HTT) and PDGFRβ leads to PDGFRβ transactivation and activation, and to PASMC proliferation and migration [32,81]. In addition, the PDGF pathway also could regulate serotonin production. Imatinib treatment led to decreased serotonin levels in iPAH patients [82]. This was further observed in the Sugen 5416 (SU5416)/CH model, a PH model associating VEGF inhibition and chronic hypoxia [82]. Reactive oxygen species (ROS) appear to be necessary for the PDGFRβ transactivation by 5-HTT in PASMC [32,81]. This positive regulation of PDGFRβ signaling is inhibited with ROS suppression.

Vascular remodeling is characterized in particular by pulmonary vascular muscularization due to PASMC proliferation. In vitro, proliferation and migration of PAH and rodent PASMC are inhibited when cells are treated with Imatinib, indicating PDGFRβ implication [3,4,78]. A small population of PASMC primed to proliferate was identified in pulmonary arterioles, located close to the muscularized/non-muscularized zone border [52]. Upon chronic hypoxia, these PASMC initiate a sequential program of dedifferentiation (SMMHC-/KLF4+/PDGFRβ+ PASMC) and redifferentiation to spread along the initially non muscularized zone. This sequential program is due to PDGFRβ activation by EC-secreted PDGF-B [83]. Increased KLF4 expressing PDGFRβ+/KLF4+ PASMC were also observed in pulmonary arteries of PAH patients [52]. PDGFRs activation also led to pulmonary smooth muscle progenitor cells differentiation into news PASMC [52,83]. PDGFRα inhibition reduced chronic hypoxia-induced proliferation and differentiation of PW1+ perivascular progenitor cells into PASMC [31]. Their role in human disease is suggested by increased numbers of PW1+ perivascular cells and by the presence of PW1+/α-SMA+ PASMC in PAH patient lungs. PDGFRβ is known to be implicated in the recruitment of pericytes which are known to be SMC progenitor cells. In PH models induced by chronic hypoxia or MCT injection, an increased NG2+/3G5+ pericyte coverage of pulmonary vessels is observed. This increased coverage is possibly due to PDGFRβ activation [84]. This coverage is also found in the lungs of PAH patients with a low proportion of α-SMA+/SM22+ pericytes in contrast to control lungs where pericytes are negative for these SMC markers.

## 5. Clinical Assessment of Therapies Targeting the PDGF Pathway in PAH Patients

Imatinib was the first TKI introduced for treatment of chronic myeloid leukemia and has become a gold standard for this pathology. Given the contribution of PDGFR to PAH and the experimental results obtained with Imatinib, clinical trials have been carried out with Imatinib with great hope. In the IMPRES study (a 24-week phase III clinical trial), Imatinib, in addition to other therapies, showed an improvement in the 6 min walk test and in hemodynamic parameters in patients with advanced PAH. However, the treated group of patients showed more treatment discontinuation due to side effects (nausea, diarrhea, peripheral edema, etc.) than the placebo group. Moreover, rare but serious side effects occurred in treated patients: heart failure (potentially related to inhibition of c-abl by Imatinib), subdural hematoma, dyspnea and syncope. During the trial extension to 204 weeks, Imatinib provided significant improvement only in few treated patients but, in rare cases, caused serious side effects that counterbalanced the positive effects observed. Thus, despite encouraging in vivo results, Imatinib did not provide sufficient benefit and safety to be used as a treatment for PAH [5,85].

Other receptors, such as tyrosine kinase (RTK) inhibitors, were assessed to target PDGFRs pathways. Nintedanib is a non-specific tyrosine kinase inhibitor, targeting PDGFRs, FGFRs and VEGFRs that is FDA approved for the treatment of idiopathic pulmonary fibrosis. Two studies showed the opposite effect of Nintedanib treatment in SU5416/CH rats. Nintedanib did not reverse PH and showed no effect on pulmonary vascular remodeling in one study but improved vascular remodeling in another study [86,87]. These contrasted effects may be explained by differences in rat strains. In fact, rat strains show dissimilar responsiveness to SU5416 and may also respond differently to treatments. Differences in the delivery route of Nintedanib could also explain these contrary effects. Nintedanib seems to reduce vascular remodeling by inhibiting EndoMT transition and PASMC proliferation [87]. In four severe PAH patients, Nintedanib failed to improve pulmonary hemodynamics and right heart function [88]. Hence, the efficacy of Nintedanib to moderate PAH is still uncertain.

Sorafenib is a combined tyrosine and serine/threonine kinase inhibitor and is FDA approved for treating carcinomas. It prevents MCT-induced PH in rats, reducing pulmonary vascular muscularization and improving cardiac function [89,90]. This multiple kinase inhibitor was well tolerated by PAH patients in a phase 1b clinical trial [91]. Add-on therapy using Sorafenib in a small cohort of severe and refractory iPAH patients had favorable effects improving symptoms [92]. 

Hence, several compounds inhibiting RTK including PDGFR have been evaluated in PAH patients. The disappointing results obtained using Imatinib has reduced hopes concerning this class of therapeutic molecules. In addition, Dasatinib—a second generation RTK inhibitor targeting PDGFR, c-kit, c-abl, arc kinases and EPHA2—was found to predispose patients to pulmonary hypertension [93]. This further raised concerns on the use of multiple kinases inhibitors. Clinicians and researchers are now preferentially assessing either more specific inhibitors or more localized treatments.

## 6. Potential Future Therapies Targeting the PDGF Pathway in PAH Patients

### 6.1. Receptor Tyrosine Kinase Inhibitors

**Figure 5:**
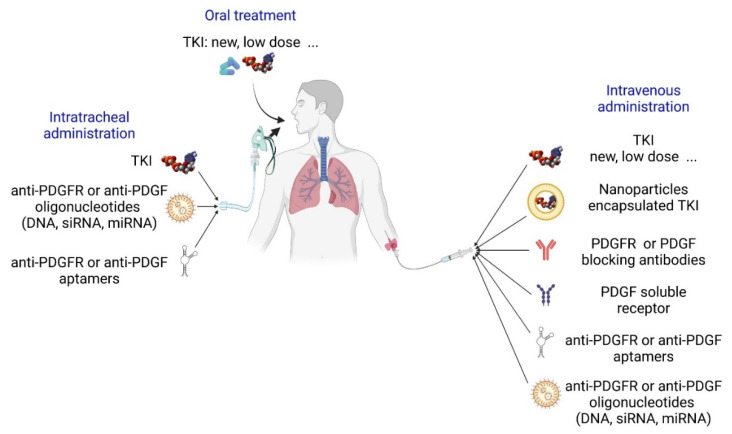
Future therapies targeting the PDGF pathway in PAH patients. Most of tyrosine kinase inhibitors (TKI) are non-specific and target several tyrosine kinase receptors. Imatinib treatment of iPAH patients showed some beneficial effects but raised safety concerns. New TKI with various specificities are being tested and could provide improvement with less adverse effects. In addition, clinical trials are ongoing to test the efficacy and safety of low doses of Imatinib administered by nebulization. TKI could also be encapsulated in different nanoparticles (liposomes, dendrimers, magnetic nanoparticles…) to target the lung. Other future therapies could involve molecules which could specifically target only one member of the PDGFR/PDGF family. Blocking antibodies are now being tested in different pathologies and could therefore be evaluated in PAH patients. Soluble truncated receptors can also serve as decoy receptors for PDGF. Other nucleotidic therapeutic molecules are also being developed. Aptamers are RNA molecules which can bind and inhibit proteins. Antisense oligonucleotides (DNA, siRNA and miRNA) are also a new avenue for disease treatment that could help reduce PDGF signaling during PAH. TKI, Tyrosine Kinase Inhibitor; siRNA, short inhibitory RNA; miRNA, microRNA.

Considering the relative RTK selectivity of Imatinib and the beneficial effects observed in some patients [94,95], the molecule is still of great interest and is tested in different conditions. One hypothesis is that targeting only the lung might reduce adverse effects, in particular on the heart, and still show improvements in vascular remodeling and pulmonary pressure. Indeed, inhalation of Imatinib powder has been proposed since it reduced preclinical studies on MCT-treated rats [96] and two clinical trials have been launched to evaluate this treatment (Figure 5). Imatinib could also be delivered intratracheally incorporated in nanoparticles [97]. Reducing the amount of Imatinib administered is also under evaluation [98] as low doses still normalized vessel muscularization in the MCT rats [99]. Three patients out of five showed improvements when receiving a low dose of imatinib for 24 weeks [100]. 

Other RTK inhibitors showed variable effects and efficacity in PH models. Moreover, the global effect on the development of PH is not necessarily only due to the inhibition of PDGFRs. Seralutinib (GB002) targets PDGFRs, c-kit and CSF-1R. It was evaluated in two experimental models: the monocrotaline/pneumonectomy model and the Sugen5416/CH model. In these models, Seralutinib reversed pulmonary vascular remodeling, improved hemodynamics parameters and upregulated BMPR2 expression [101,102]. A phase 2 trial (TORREY) for Seralutinib has been launched recently to assess pulmonary vascular resistance and 6MWD (6-Minute Walking Distance) improvement [103]. Masitinib, has a similar target selectivity as Imatinib and improved right ventricle contractility and pulmonary medial hypertrophy in MCT-injected rats [104]. BIBF1000 is another small molecule tyrosine kinase inhibitor close to Nintedanib which also targets PDGFRs, VEGFRs and FGFRs and shows strong anti-fibrotic effects. BIBF1000 inhibited in vitro hypoxia-induced rat PASMC proliferation and migration and reversed PH development in the MCT+pneumectomy rat model decreasing the pulmonary vascular pressure, right ventricular systolic pressure (RVSP), right ventricular hypertrophy, medial wall thickness, vascular occlusion scores and lung fibrosis [105]. Sunitinib inhibits PDGFRs, VEGFRs, c-kit, FLT3, CSF-1R and RET and is FDA approved for the treatment of several cancers. Sunitinib showed limited effects in reversing MCT-induced PH. It decreased PDGFRβ mRNA level and medial wall thickness of fully muscularized vessels but did not improve right ventricular hypertrophy nor did it reduce pulmonary arterial muscularization [99]. Toceranib is an inhibitor targeting PDGFRs, VEGFRs and c-kit. In the MCT-induced PH model, Toceranib did not decrease the RVSP. Only a high dose of Toceranib partially reversed the right ventricular hypertrophy. Toceranib decreased PDGFRβ mRNA level but had a low impact on vascular remodeling with a decrease in medial wall thickness but no effect on vessel muscularization [90]. More recent TKI also needs to be evaluated (such as Ripretinib, etc.). 

Due to their non-selectivity and to the broad range of RTK expression, RTK inhibitors toxicity is challenging although patients present sometimes unexpected positive evolution in complex contexts [106]. Several possible ways to reduce these toxic effects are studied. As tested for Imatinib, direct intratracheal administration is a promising approach (Figure 5). Targeting their delivery could be achieved using nanostructures such as nanoparticles, magnetic nanoparticles, liposomes, dendrimers, exosome [107]. However, designing safe and efficient nanostructures is still a challenge. Production of new TKIs with different mechanisms of action (e.g., Ripretinib) or with higher selectivity will also be an important step (see specific inhibitors). Combination therapies could also be of interest. A low dose combination of Imatinib with Rapamycin attenuated PH, reducing RVSP, right ventricle hypertrophy and vessels muscularization, in MCT and SU5416/CH rats [108]. 

### 6.2. Specific PDGF/PDGFR Inhibitors

Besides RTK inhibitors, another approach is to directly target each of the members of the PDGFR pathways. PDGFRα and β can be specifically inhibited using blocking antibodies (Figure 5). A PDGFRα blocking antibody (Olaratumab) was tested for tumor treatment in phase 2 and 3 clinical trials [109,110]. However, its development was stopped since it did not reach the primary endpoints as a cancer drug. The important role of PDGFRα in controlling pulmonary vessels muscularization and the beneficial effect of anti- PDGFRα therapy in reducing chronic hypoxia-induced vascular remodeling suggest that PDGFRα alone could be targeted in patients [31]. A human PDGFRβ-blocking antibody was produced (IMC-2C5) but has not been evaluated in patients [111]. Blocking antibodies against PDGF isoforms could be also effective. For instance, blocking PDGF-CC using the human antibody 6B3 showed similar beneficial effects as blocking PDGFR with Imatinib in a mouse model of brain–blood barrier injury [112]. Hence, testing the effect of trapping specific PDGFs on PH development will be stimulating. In this regard, soluble extracellular domains of PDGFR are also of potential use. They have been known for a long time and effectively block PDGF binding to their receptor [113]. 

Other ways to specifically inhibit the PDGFRs are being developed with some of them under clinical assessment (Figure 5). RNA aptamers are promising tools. They are single stranded structured nucleic acid ligands (DNA or RNA) that bind to their target similarly to antibodies with a high specificity and a high affinity. They show thermal and chemical stability and are obtained through simple chemical synthesis. Anti-PDGFRs and PDGFs aptamers have been produced and need to be evaluated. Targeting PDGF-B with an aptamer reduced perinatal PH and vascular remodeling in lambs with chronic intrauterine PH [114]. Interestingly, E10030 is an anti-PDGF-B pegylated aptamer that has been used in clinical trials for macular degeneration. Specific downregulation of PDGF and PDGFR expression can be achieved by antisense oligonucleotides (Oligodeoxynucleotides, siRNAs, miRNAs). They bind specifically to their target RNA—mostly mRNA, but other RNAs can also be targeted—to induce its degradation or inhibit its translation. They can be chemically modified to enhance their stability and incorporated into nanostructures for better delivery. RNA-targeted drugs are under development, and some have already been approved for several pathologies [115]. Ongoing research mostly focuses on delivery through inhalation, whereas others aim at selectively target lungs and other organs using nanoparticles with specific classes of lipids. Several studies have demonstrated PH improvement in experimental models with miRNAs or siRNAs administration [116,117]. Recently, intratracheal administration of nanoparticles containing PDGF-B siRNAs was shown to prevent chronic hypoxia-induced pulmonary vessel muscularization, RVH and PH in mice [70]. The effects of antisense oligonucleotides-induced inhibition of PDGFs or PDGFRs on PH development remain to be determined.

## 7. Conclusions

PDGF is a major regulator of pulmonary vascular remodeling. Multiple experimental data, together with the encouraging results of Imatinib clinical trial on pulmonary vascular resistance, suggest that inhibiting members of the PDGF family, either together or separately, could be beneficial for PAH patients. The cruel lack of therapeutic options urges to test new delivery techniques or doses of available drugs, to assess drugs under development and to develop new molecules. The important contribution of PDGF to multiple pathological and physiological processes explains that it is one of the most highly studied pathways. Hence, the large panel of PDGF/PDGFR targeting compounds that are currently being assessed or that could be assessed in the near future allows some hope to find add-on therapeutic tools and reduce side effects. Indeed, the recent development of adenoviral or RNA vaccines shows that new strategies may be very efficient and groundbreaking. Precisely understanding of the spatiotemporal actions of the various PDGF will also help to direct the anti-PDGF therapy and reduce. Finally, a full in-depth comprehension of the molecular basis of specific downstream intracellular signals and cellular responses for each member of the PDGF family in each cell type is mandatory to facilitate identification of new potential targets. In this regard, the use of developing technologies such as organoids or organ-on-chip will be of particular interest to model cellular interactions and regulations and study the role of PDGFs and PDGFRs and the consequences of their inhibition.

## Data Availability

Not applicable.
